# Evaluation of Neurosecretome from Mesenchymal Stem Cells Encapsulated in Silk Fibroin Hydrogels

**DOI:** 10.1038/s41598-019-45238-4

**Published:** 2019-06-19

**Authors:** Yolanda Martín-Martín, Laura Fernández-García, Miguel H. Sanchez-Rebato, Núria Marí-Buyé, Francisco J. Rojo, José Pérez-Rigueiro, Milagros Ramos, Gustavo V. Guinea, Fivos Panetsos, Daniel González-Nieto

**Affiliations:** 1 Center for Biomedical Technology, Universidad Politécnica de Madrid, Madrid, Spain; 2 Departamento de Tecnología Fotónica y Bioingeniería. ETSI Telecomunicaciones, Universidad Politécnica de Madrid, Madrid, Spain; 3 Departamento de Ciencia de Materiales. ETSI Caminos, Canales y Puertos, Universidad Politécnica de Madrid, Madrid, Spain; 4 Biomedical Research Networking Center in Bioengineering Biomaterials and Nanomedicine (CIBER-BBN), Madrid, Spain; 5 Neurocomputing and Neurorobotics Research Group: Faculty of Biology and Faculty of Optics, Universidad Complutense de Madrid., Madrid, Spain; 6 Brain Plasticity Group. Health Research Institute of the Hospital Clínico San Carlos (IdISSC), Madrid, Spain; 7 Present Address: GReD, UMR CNRS 6293 – INSERM U1103 – Université Clermont Auvergne, Faculté de Medicine, Clermont-Ferrand, France

**Keywords:** Biomaterials - cells, Drug delivery

## Abstract

Physical and cognitive disabilities are hallmarks of a variety of neurological diseases. Stem cell-based therapies are promising solutions to neuroprotect and repair the injured brain and overcome the limited capacity of the central nervous system to recover from damage. It is widely accepted that most benefits of different exogenously transplanted stem cells rely on the secretion of different factors and biomolecules that modulate inflammation, cell death and repair processes in the damaged host tissue. However, few cells survive in cerebral tissue after transplantation, diminishing the therapeutic efficacy. As general rule, cell encapsulation in natural and artificial polymers increases the *in vivo* engraftment of the transplanted cells. However, we have ignored the consequences of such encapsulation on the secretory activity of these cells. In this study, we investigated the biological compatibility between silk fibroin hydrogels and stem cells of mesenchymal origin, a cell population that has gained increasing attention and popularity in regenerative medicine. Although the survival of mesenchymal stem cells was not affected inside hydrogels, this biomaterial format caused adhesion and proliferation deficits and impaired secretion of several angiogenic, chemoattractant and neurogenic factors while concurrently potentiating the anti-inflammatory capacity of this cell population through a massive release of TGF-Beta-1. Our results set a milestone for the exploration of engineering polymers to modulate the secretory activity of stem cell-based therapies for neurological disorders.

## Introduction

In recent years, an unprecedented amount of scientific work has pushed the development of cell-based strategies to protect and repair the injured brain^1^. Cell therapy-based treatments have been tested in patients with stroke, traumatic brain injury and neurodegenerative disorders such as Alzheimer’s and Parkinson’s diseases. However, clinical outcomes have not reached the anticipated and strong success of preclinical trials. Patient-to-patient variability and lack of standardized procedures might have contributed to this adverse scenario. Immune-compatibility concerns, dosing, administration route and cell manipulation might also explain this limited translatability^2^. In particular we have ignored most of the neurotherapeutic cues related to the beneficial aspects of cell transplantation.

A significant amount of evidences supports that the neuro-protective and neuro-repairing benefits of many stem cells and progenitors mainly rely on their capability to secrete small molecules and different factors with neuroprotective, anti-inflammatory and angiogenic activities; these factors are together termed the “secretome”^3^. It is also accepted that many of these secreted factors might stimulate endogenous neurogenesis^3^, which is an interesting aspect to explore since the formation of newborn neural cells in the adult brain is inefficient and declines with age^4,5^. This neuro-secretome activity has been found in different multipotent (neural, mesenchymal and hematopietic stem cells) and pluripotent (embryonic and induced by reprogramming) stem cells. Particularly interesting is the functional secretome of mesenchymal stem cells (MSCs), which exhibit both direct paracrine release of factors and release by extracellular vesicles or exosomes^3,6^

Today MSCs, which have the capacity for self-renewal and differentiation into one or more specialized cell types, constitute the most frequently used cell population in regenerative medicine. The immunosuppressive character of MSCs which is based on their unique cross-talk with hematopoietic cells and the poor immunogenic properties of MSCs are ideal for autologous and allogenic transplantation. In addition, no evidence of tumorigenicity has been attributed to their therapeutic use. Apart from cord blood, dental pulp and fat, multipotent MSCs are more prevalently found in the bone marrow where they provide signaling for hematopoietic stem cell physiology and hematopoietic regeneration after bone marrow ablation^7^. MSCs have been extensively used in the majority of cell therapy studies involving patients. For example, a phase III randomized clinical trial using MSCs to treat anal fistulas in patients with Crohn’s disease has achieved significant benefits^8^. Graft-versus-host disease has also been treated with MSCs in children^2^. In the context of the nervous system, several preclinical studies have set a strong precedent for the intense exploration of the neurotherapeutic ability of MSCs^9,10^, which have been related with anti-inflammatory and immunosuppressive effects, reducing apoptosis and promoting angiogenesis, neurogenesis and synaptogenesis^11^. In patients with cerebral damage, MSCs have been transplanted with promising outcomes^12^. It is expected that the development of MSCs-based technologies represents an important challenge to diminish global burdens in cerebrovascular and neurodegenerative diseases.

Although animal studies have encouraged the use of cell-based therapies in the clinical setting, cerebral transplantation of undifferentiated and mature cells has resulted in poor engraftment^13–16^. Long-term survival of the grafted cells is desirable in the majority of biomedical applications. However, the engraftment of MSCs is limited in the host brain and a strong declining in MSCs survival has been reported in different brain areas as soon as one week after implantation^16–18^. Different biopolymers have been employed to provide innocuous scaffolds to favor survival, retention and integration of therapeutic cells in brain tissue^19^. Hydrogel-based biomaterials have been used to mimic the *in vivo* microenvironment of soft tissues such as the brain to fill completely amorphous cavities resulting from injury, as in stroke or physical brain trauma. The different hydrogels can be tuned to adjust porosity, gelation time and degradation rate to provide tailorable biomaterials for nervous tissue reconstruction. These biomaterials might potentiate cell survival leading to persistent therapeutic effects.

Silk fibroin (SF) is an adaptable natural biomaterial that has been used for multiple applications in the area of biomedicine^20,21^. Among the best properties of silk are its inertness and null immunogenicity compared to other natural materials. Its structural, biological and mechanical properties can be engineered to the target tissue, making silk a versatile biomaterial. SF can be manufactured into different formats including fibers, films or gels. Recently, we have found that this biomaterial is well tolerated by the central nervous system^22^. In addition, SF increases mesenchymal stem cell engraftment promoting neuroprotection and brain plasticity that sustain functional recovery after stroke^23^. A nice work has also recently confirmed the good compatibility of SF with the ischemic brain in rats^24^. This biomaterial implanted in the stroke cavity promoted a favorable environment that supports endogenous cellular mechanisms after brain injury^24^.

The interaction of MSCs with different natural or synthetic biomaterials of different compositions and formats has been explored in many studies; however, much less is known about the effect of different polymers such as SF on the regulation of the MSCs secretome, which is the functional correlate that sustains the neurotherapeutic ability of MSCs^3,25^. Consequently, we test basic aspects of culturing MSCs engrafted in 3D fibroin hydrogels, including their secretome capacity. In addition, we discriminate the relative influence of spatial confinement and chemical environment in the cells by studying the survival and proliferation of MSCs cultures on 2D fibroin films.

## Material and Methods

Other methods can be found in Supplementary Material (available on the Scientific Reports Web site).

### Silk fibroin extraction and preparation of hydrogels and films

SF was obtained from *Bombyx mori* cocoons and processed as we have previously described^23^. Cocoons, generously provided by Professor Jose Luis Cenis (IMIDA, Murcia, Spain), were cut into pieces and degummed in 0.02 M sodium carbonate solution to remove sericin. After degumming fibroin fibers were washed with distilled water and dried overnight. Dry fibers were dissolved in 9.4 lithium bromide under continuous shaking and the solution was dialyzed for 48 hours against water, centrifuged to remove impurities, frozen (−80 °C) and subsequently lyophilized (LyoQuest, Telstar). Fibroin hydrogels were fabricated from lyophilized SF by mixing it with Dulbecco’s Modified Eagle’s Medium (DMEM) at 2% (w/v) concentration as explained in detail elsewhere^22,23^.

Fibroin films were produced from 2, 4, 6, and 8% (w/v) fibroin solution in 1,1,1,3,3,3-hexafluoro-2-propanol (HFIP, Sigma Aldrich; Cat# 105228) by casting 200 μl of the filtered (Sterile Syringe Filter 0,2 µm, VWR) solution into well plates (BioLite 24 Wll Multidish, Thermo Scientific) in a concentration of 3.2 μg/cm^2^. After polar solvent evaporation SF films were treated with serial solutions of ethanol (80% for 60 min; 70% 30 min; 50% 10 min and finally 20% during 10 min) to cause protein insolubilization (films). Finally, the ethanol solution was removed and films were completely dried overnight. Before use the films were repeatedly washed with distilled water and stored at 4 °C.

### Mechanical characterization

The mechanical properties of SF hydrogels were assayed under unaxial unconfined compression tests as previously described^22^. SF solutions (pre-gel state) were deposited into cylindrical molds (10.7 mm in diameter) allowing the solution to gel at room temperature. After 36 hours the gels were cut in approximately 10 mm height cylinders and placed between two parallel plates adapted to an Instron 5543A machine. The force applied was measured with an electronic balance (Precisa XT 220A) and the compression speed was fixed at 0.03 mm/s. The cross-sectional areas were used to compute stress-strain curves from force-displacement. The modulus of elasticity as a function of SF concentration was obtained by calculating the slope of the straight-line portion of the stress-strain curves.

### Monitoring the gelation process

SF gelation was induced by sonication as we have previously described^22^. Briefly, SF dissolved at 2% (w/v) in complete medium (DMEM supplemented with 10% of fetal bovine serum, 1% penicillin/streptomycin and 2 mM L-Glutamine) or in phosphate buffer saline (PBS) without Ca^2+^/Mg^2+^ was filtered through 0.2 μm filter. The filtered solutions were sonicated in Eppendorf tubes with a Branson 450 Sonifier coupled to a 3.17 mm diameter Tapered Microtip, delivering ultrasonic vibrations at 20 kHz for 1–10 min and sonication amplitudes with ranges of 5–15%. Turbidity changes were monitored at short (1 hour) and long (48 hours) time intervals using a UV/Vis spectrophotometer (Halo RB-10 - Dynamica Scientific Ltd). Alternatively, quasi-static behaviour identification was performed with an Instron 5543 device coupled to a blunt-end tip (1 mm diameter). The tip was immersed in the pre-gel solution at 0.03 mm/min speed. The magnitude of load was recorded over time after immersion with a laboratory balance (Model XT220A, Precisa Gravimetrics AG).

### Isolation of mouse bone marrow mesenchymal stem cells, enrichment and establishment of cell cultures

Mouse mesenchymal stem cells (MSCs) were obtained from fresh bone marrow, extracted and processed as previously described^7^. The sacrifice of animals was carried out after ethical approval from Universidad Politécnica de Madrid, according to the Spanish Regulations for animal experimentation (Laws 53/2013, 178/2004) and under the approval of Community of Madrid with authorization number PROEX 393/15. The experimental procedures were performed according to the ARRIVE guidelines. The CD-1 mice were sacrificed with CO_2_ followed by cervical dislocation. Flat (pelvis) and long bones (femur and tibia) were harvested under sterile conditions in a laminar flow cabinet (Labgard class II Biological safety cabinet, ES). The bones were crushed in a porcelain mortar with cold PBS at 4 °C (Gibco® life technologies). The cell content after trituration was filtered through a 70 μm-cell strainer (BD bioscience) and centrifuged (Hettich Rotina 380 R) at 1500 rpm for 5 min at 4 °C. The supernatant was discarded, and the resulting pellet was resuspended with 10 ml of lysis solution (BD Pharm Lyse ™) for 5 min at room temperature to remove the red blood cells. After a second centrifugation (1500 rpm for 5 min), the new pellet was resuspended in expansion medium consisting of Iscove’s Modified Dulbecco’s Medium (IMDM, gibco® life technologies) supplemented with 20% mesenchymal serum (Stem Cell Technologies, Cat#05502), 100 U/ml penicillin/0.1 mg/ml streptomycin (gibco® life technologies), 100 μM 2-Beta-mercaptoethanol (Fisher Scientific), 2 mM L-glutamine (gibco® life tecnologies), 10 ng/ml human platelet-derived growth-factor (hPDGF-BB; Peprotech®; Cat# 100-14B), and 10 ng/ml recombinant mouse epidermal growth factor (rmEGF; Peprotech®; Cat# 315-09). Cells were seeded at a concentration of 6.5 × 10^5^/cm^2^ in ventilated culture flasks (BioLite 75 cm2 Flask Vented, Thermo scientific) previously treated with fibronectin (Sigma aldrich, Cat # F4759) at a concentration of 4 μg/cm^2^ for 3 hours at 37 °C. After 72 hours of incubation (37 °C, 5% CO_2_, 95% air and 100% humidity), half of the culture medium was replaced by fresh expansion medium. Hemi-depletion was carried out weekly. Although highly enriched with stem cells, the MSCs cultures contain a polyclonal mixture of mesordermal cells (stem cells, progenitors, precursors). As described above, the isolation and initial *ex vivo* expansion of MSCs was performed in expansion medium containing EGF and PDGF as mitogen factors^26,27^ in order to preserve the more primitive bone marrow mesenchymal precursors, self-renewal capacities and therapeutic potential of undifferentiated MSCs^7^. Once the MSCs cultures were free of adhered macrophages, typically 4–5 passages after bone marrow extraction and initial seeding, the regular maintenance of the MSCs was performed in complete medium consisting of DMEM supplemented with 10% fetal bovine serum (FBS), 1% penicillin/streptomycin and 2 mM L-Glutamine. All *in vitro* studies were performed in passage numbers 5 to 15.

### Cell viability

Cell viability in SF gels was explored by mixing a pellet containing nearly 1 × 10^5^ MSCs in ~700 μl of a sonicated SF solution still in a pre-gel state. The whole mixture was deposited into a sterile cylindrical mold. The SF solution was prepared by dissolving 2% of SF in complete MSCs medium. Once the gelation was complete the cylindrical molds were cut into transverse sections. The sections were introduced into 24 well plates, covered with 500 μl of complete medium and maintained in culture for two weeks. The medium was replaced every three days. Cell viability was assayed by staining with Calcein-AM (eBioscience; Cat# 65-0853) and propidium iodide (PI, Sigma Aldrich; Cat# P4170) for 20–30 min at 37 °C. Calcein and PI were used at 5 µM and 1.5 µM, respectively. Viability was estimated by counting the number of Calcein- and PI-positive cells with the help of imageJ 1.52i (NIH). At least 4 optical fields were taken and analyzed per sample. Identification and imaging of calcein (excitation/emission at 495/515)- and PI (excitation/emission at 493/636)-positive cells was performed under a fluorescence microscope (Leica DMI3000, Nussloch, Germany) coupled with a Leica DFC340FX camera.

Short- and long-term viability of MSCs over SF films and plastic were assessed in 24 well plates (BioLite, Thermo Scientific). Approximately 20 × 10^3^ MSCs were seeded over the different biomaterials. After 48 hours in complete medium, 0.1 µg/ml of mitomycin C (Sigma Aldrich; Cat# M0503) was added for 12 hours (37 °C) to induce a prolonged quiescent state, and the MSCs cultures were maintained in the same wells for several weeks. At each particular time point, the cells were incubated with PI for 20–30 min (37 °C). The stained cells were washed twice with PBS, fixed with paraformaldehyde (4%), washed again with PBS and filtered through a 100 μm cell strainer. In these assays, fresh MSCs detached 24 hours after initial seeding were used as a control for viability. Incubation with dimethyl sulfoxide was used as a control for death. Flow cytometry was carried out in a BD FACSCalibur (Becton, Dickinson and Company) on the FL2 channel (excitation at 488 nm and emission at 585 nm). The acquisition of events were performed upon OS 9.2.2, CellQuest Pro 4.0.2 software and the analysis of alive/death cell events using FlowJo software (FlowJo® v10.2, LLC, Ashland, Oregon).

### Adhesion studies

Adhesion studies of MSCs were performed by incubating 20 × 10^3^ cells on uncoated culture plastic (TCP) (multi-dish 24 wells; BioLite, Thermo Scientific) or TCP coated with SF films, fibronectin (LifeTechnologies cat# 33010-018) and laminin (Corning cat# 354232). Fibronectin and laminin were deposited on TCP in PBS at 4 μg/cm^2^ and 2.5 μg/cm^2^ respectively. After 2 h of incubation (37 °C) the wells were rinsed with water, dried and stored at 4 °C until further use. Early contact of MSCs to the different substrates was examined by conventional stick-wash assays at different time point after initial seeding. To quantify the strength of cellular adhesion inverted centrifugation assays were performed as previously described with some modifications^28^. SF films, fibronectin and laminin coatings were prepared on circular borosilicate glasses (12 mm in diameter) that were placed into multi-dish 24 wells plates. For SF films, glass coverslips were previously pre-treated with poly-L-lysine (1.5 µg/cm^2^, Sigma Aldrich; Cat# A-005-M) during 1 hour at 37 °C. A total of 20 × 10^3^ MSCs were seeded on the glass coverslips uncoated or coated with the different biomaterials tested. Fibronectin and laminin were deposited at the same concentration used for TCP wells. After 48 hours of incubation the coverslips were carefully removed from the wells, flipped upside down and placed over Eppendorf tubes filled with PBS until a positive meniscus was generated. The tubes were carefully sealed with plate sealing tape to avoid any air bubbles. Centrifugation protocols were conducted for 5 min at 1, 2, 8, 50 and 233 relative centrifugal forces (r.c.f.; Hettich Rotina 380 R centrifuge). After centrifugation the adhered cells on the different substrates were recovered by trypinization and counted. The percentage of adherent cells was calculated by: 100 × [adherent cells after centrifugation (“output”)/total cells number seeded initially on the coverslips (“input”)].

### Secretion analysis

The concentration of stromal cell-derived factor 1 (SDF-1), brain-derived neurotrophic factor (BDNF), vascular endothelial growth factor (VEGF) and transforming growth factor beta-1 (TGF-Beta-1) was determined in conditional medium from non-encapsulated or encapsulated MSCs in SF hydrogels. After 48 hours in culture (at 37 °C with 5% CO_2_) the medium was removed and cells were incubated with fresh medium with or without tumor necrosis factor-alpha (TNF-alpha, 50 ng/ml, Preprotech 315-01A). This TNF-alpha concentration was selected based on previous studies that examined the involvement of TNF-alpha modifying the secretion of several factors such as VEGF or glial cell derived neurotrophic factor (GDNF) from bone marrow MSCs^29,30^. After 12 hours the medium was collected and processed for enzyme-linked immunosorbent assay (ELISA) according to the manufacturer’s protocols (catalogue numbers: MX120, DBNT00, MMV00 and MB200 for SDF-1, BDNF, VEGF and TGF-Beta-1 respectively, R&D Systems, Inc.). The optical density was measured at 450 nm with an absorbance microplate reader (ELx808, Biotek). Values were normalized with respect to the total number of cells in every group and dish.

### Statistical analysis

Statistical analyses were conducted using SigmaPlot (Systat, Germany). Data are showed as mean ± standard errors of the means (S.E.M.). Significant differences between groups were analyzed through different statistical models described in every corresponding figure legend. For example analysis of variance (ANOVA) was used to analyze statistical differences between groups in the adhesion, proliferation and cell distribution studies or to examine cell viability inside the hydrogels across time after encapsulation. Student’s t-test for independent samples was used to examine significant differences between groups in the secretome analysis. Statistical significance was considered for probabilities of the null hypothesis at p < 0.05.

## Results

**Figure 1:**
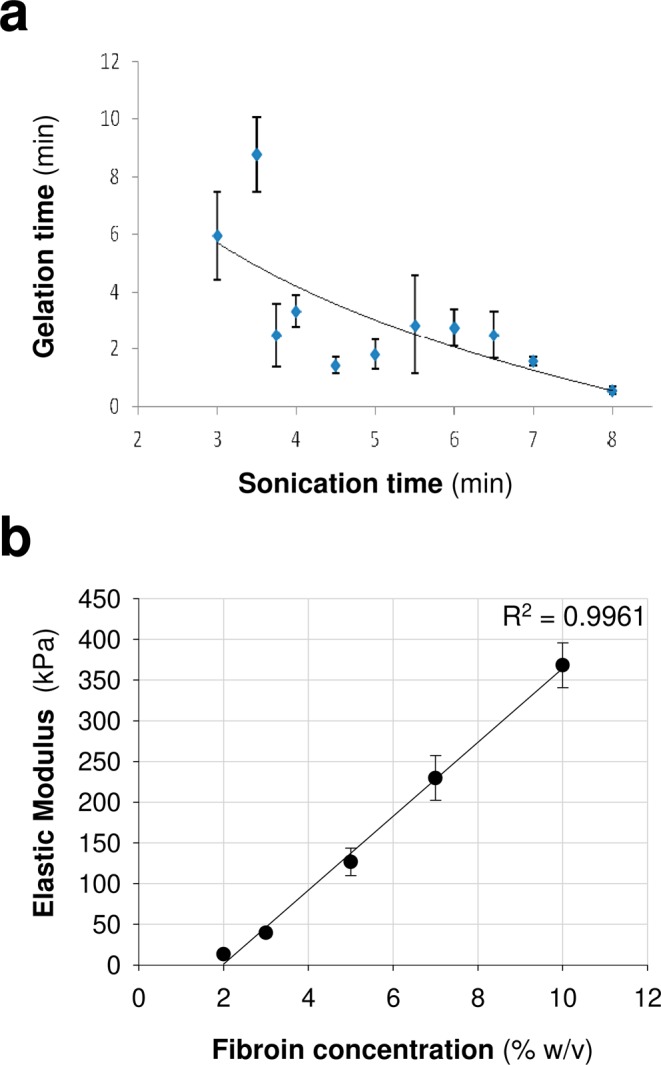
Characterization of silk fibroin hydrogels. (**a**) Analysis of the influence of sonication time on gelation time. (**b**) Stiffness of the silk fibroin hydrogel as a function of fibroin concentration. The data in a and b are shown as mean ± the SEM with a minimum of 3–7 samples analyzed per variable (sonication time or fibroin concentration).

We previously developed an *in situ*, gelling, SF hydrogel in which delayed polymerization was induced by ultrasound sonication^22^. This approach formed hydrogels *in vivo* after implantation of this biomaterial as a solution (pre-gel state) in the brain, facilitating the incorporation of cells and drugs/molecules before gelation. For cell encapsulation and subsequent implantation, the two most important characteristics of hydrogels are gelation time and the mechanical properties of the hydrogel. Gelation occurs typically within a time lapse of minutes after sonication. This allows us to incorporate the cells when the sonicated solution has enough viscosity to stop cells from settling to the bottom but is still fluid to avoid damaging the gel structure during implantation. In addition, gelation time after sonication can be modified by varying the duration of the sonication step. Figure 1a shows the relationship between gelation and sonication time, from where it is apparent the inverse dependence between these magnitudes. The relatively large scattering in the gelation time is probably related with the metastable character of the fibroin solution^22^. Gelation proceeds through the formation of β-nanocrystals between different fibroin molecules that is induced by the energy conveyed through the ultrasonic waves. In this regard, the initial condition of the proteins in the metastable solution is likely to influence the overall gelation process and justifies the scatter in the gelation times observed in experiments performed under the same processing conditions. Figure 1b shows the stiffness of the gel as a function of fibroin concentration. In contrast to the gelation time, the stiffness of the gel reaches an equilibrium value after a period of approximately 1 day^22^. From Fig. 1b it is apparent that there exists a linear relationship between stiffness and fibroin concentration in the solution in the range between 2% and 10% concentration. This dependence allows tailoring the mechanical properties of the gel to adapt them to their intended uses. In particular, it is assumed that the most interesting hydrogels for central nervous system applications are those with 2–3% fibroin concentration, since these values match the stiffness of brain tissue. In this context, our group previously demonstrated the biocompatibility of SF hydrogels with brain function^22^ and reported that the intracerebral transplantation of MSCs encapsulated in SF hydrogels enhanced the MSCs survival and engraftment, promoting functional recovery in a stroke mouse model^23^. These *in vivo* studies were performed using SF solutions with a polymer concentration of 2%. It has been previously shown that the viability of MSCs within SF hydrogels decreases drastically in 4–6% polymer concentrations^31,32^ with stiffness ranges that possibly are not adequate for intracerebral applications. Consequently, gels with a fibroin concentration of 2% were used in this study, to encapsulate cells and examine their neurosecretome activity.

**Figure 2:**
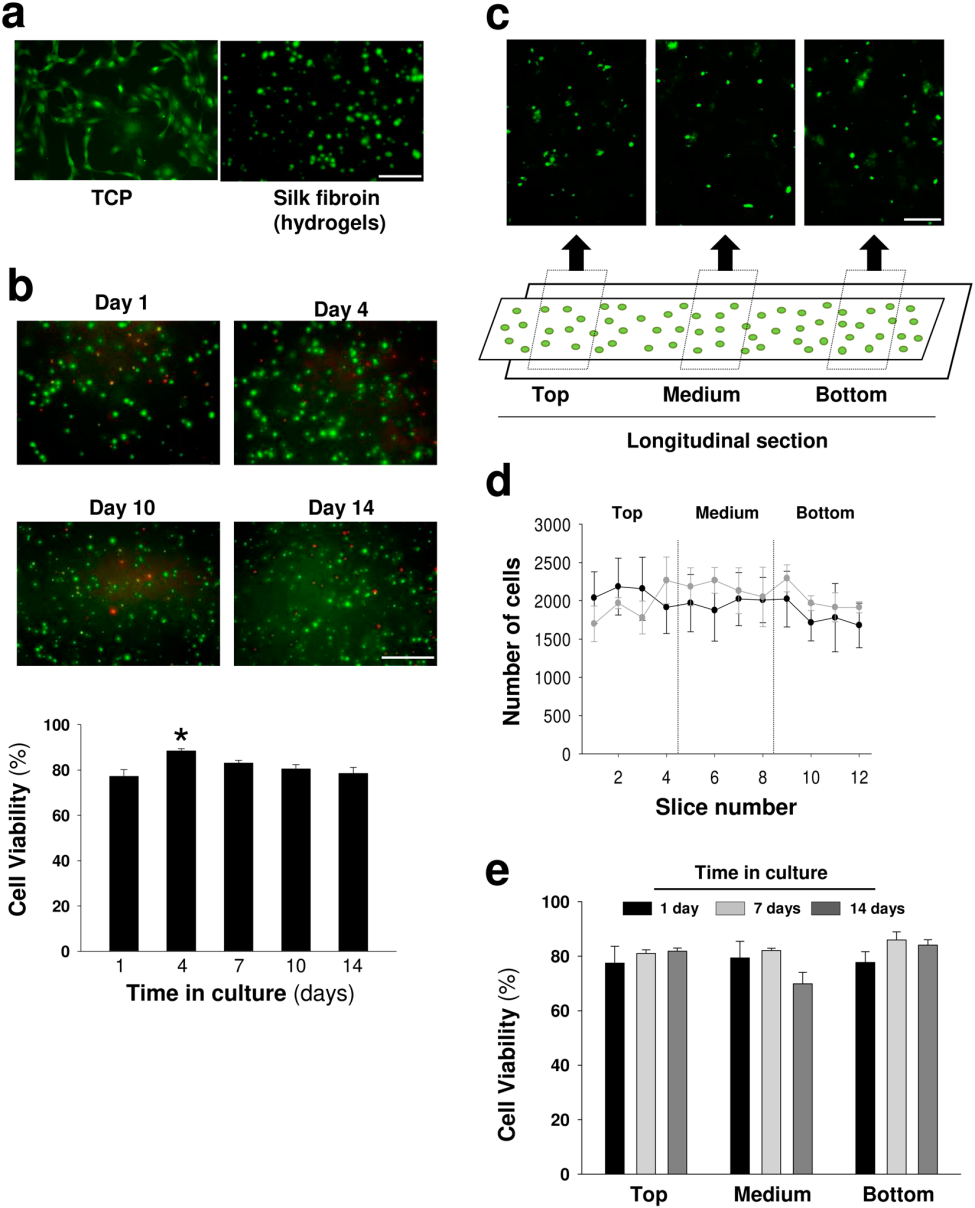
Viability and geometric distribution of mesenchymal stem cells encapsulated in silk fibroin hydrogels. (**a**) Representative images of calcein (pseudocolor green)-positive MSCs seeded on plastic (TCP) or encapsulated in silk fibroin hydrogels (Scale bar: 100 μm). (**b**) Images of calcein (green)- and propidium iodide (red)-positive MSCs at different time points after encapsulation in silk fibroin hydrogels (Scale bar: 100 μm). Below, percentage of viability inferred from the calcein/propidium iodide (alive/dead) ratio. The data are shown as mean ± the SEM with three different hydrogels per day analyzed and counted at least 300 events per hydrogel (One-way ANOVA followed by Tukey’s test; *p < 0.05). (**c**) Representative images of MSCs encapsulated in cylindrical hydrogel molds across the longitudinal axis. Different fields in the lateral (top and bottom) and central regions are shown (Scale bar: 200 μm). (**d**) Total cell counts in 12 sections across the cylindrical molds using the methodology described in the material and methods section. Cells were counted at 2 and 4 weeks after encapsulation (black and gray circles, respectively). No differences were observed between the groups (two or four weeks), suggesting that proliferation was strongly limited inside the hydrogel. The data are shown as mean ± the SEM of three independent experiments with 9 samples per section and time point after encapsulation. (**e**) Percentage of viability in different areas of cylindrical hydrogel molds. The data are shown as mean ± the SEM with three different hydrogels analyzed at different time points after encapsulation.

**Figure 3:**
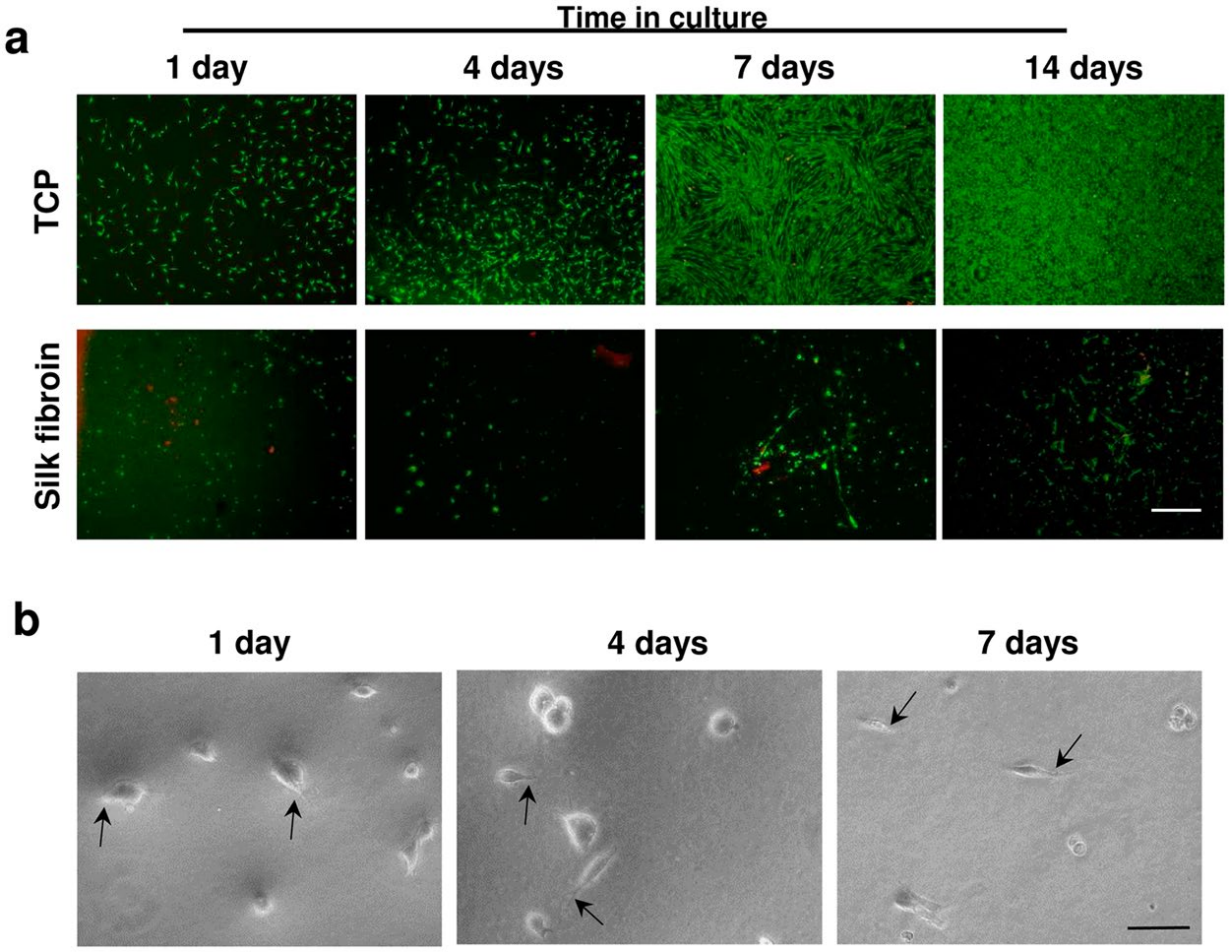
Viability of mesenchymal stem cells cultured on the top of silk fibroin hydrogels. (**a**) Representative images of calcein (green)- and propidium iodide (red)-positive MSCs at different time points after seeding on the top of plastic (TCP) or silk fibroin (2%) hydrogels (Scale bar: 500 μm). (**b**) Representative images of MSCs adhered on the top of silk fibroin hydrogels (Scale bar: 50 μm). Although the survival of MSCs was drastically reduced, some cells could be visualized exhibiting attachment and extending cellular processes (black arrows).

We performed a set of experiments to test the compatibility of this hydrogel format and polymer concentration with MSCs growth and function. We first examined the multipotent ability of MSCs to differentiate into osteoblasts, adipocytes and chondrocytes (Supplementary Fig. S1) since this assay is considered the “gold standard” for defining MSCs. As shown in Fig. 2a, most MSCs integrated into SF hydrogels lost the typical spindle-shaped fibroblast-like characteristic of MSCs **(**Supplementary Fig. S1**)**. This lymphoblast-like aspect of MSCs inside the hydrogels, typical of cells growing in suspension, is indicative of relevant cytoskeletal architecture abnormalities in MSCs associated with impaired cellular adherence. Despite these changes in cell morphology, the MSCs survived in the interior of hydrogels during the two weeks analysis with survival rates of 80–90% **(**Fig. 2b**)**. Although a slightly significant higher viability was observed on day 4, this effect was not reproduced at later points. In addition, the MSCs were equally distributed across the hydrogel polymer, and we did not detect differences in cell viability at different hydrogel locations (Fig. 2c–e**)**. Although the MSCs survival was not impaired within the hydrogel microstructure, the number of surviving cells remained constant during the 2–4 weeks analysis, suggesting that cell proliferation is abrogated inside the hydrogel (Fig. 2d). By contrast, in the interval of two weeks of analysis the adhesion and growth of MSCs on the top of 2% hydrogels was drastically reduced (Fig. 3). However, some adherent cells with fibroblastic morphologies were found on the top of these hydrogels, an observation that contrasted with the spherical morphology of MSCs residing in the interior of hydrogels (Fig. 2).

**Figure 4:**
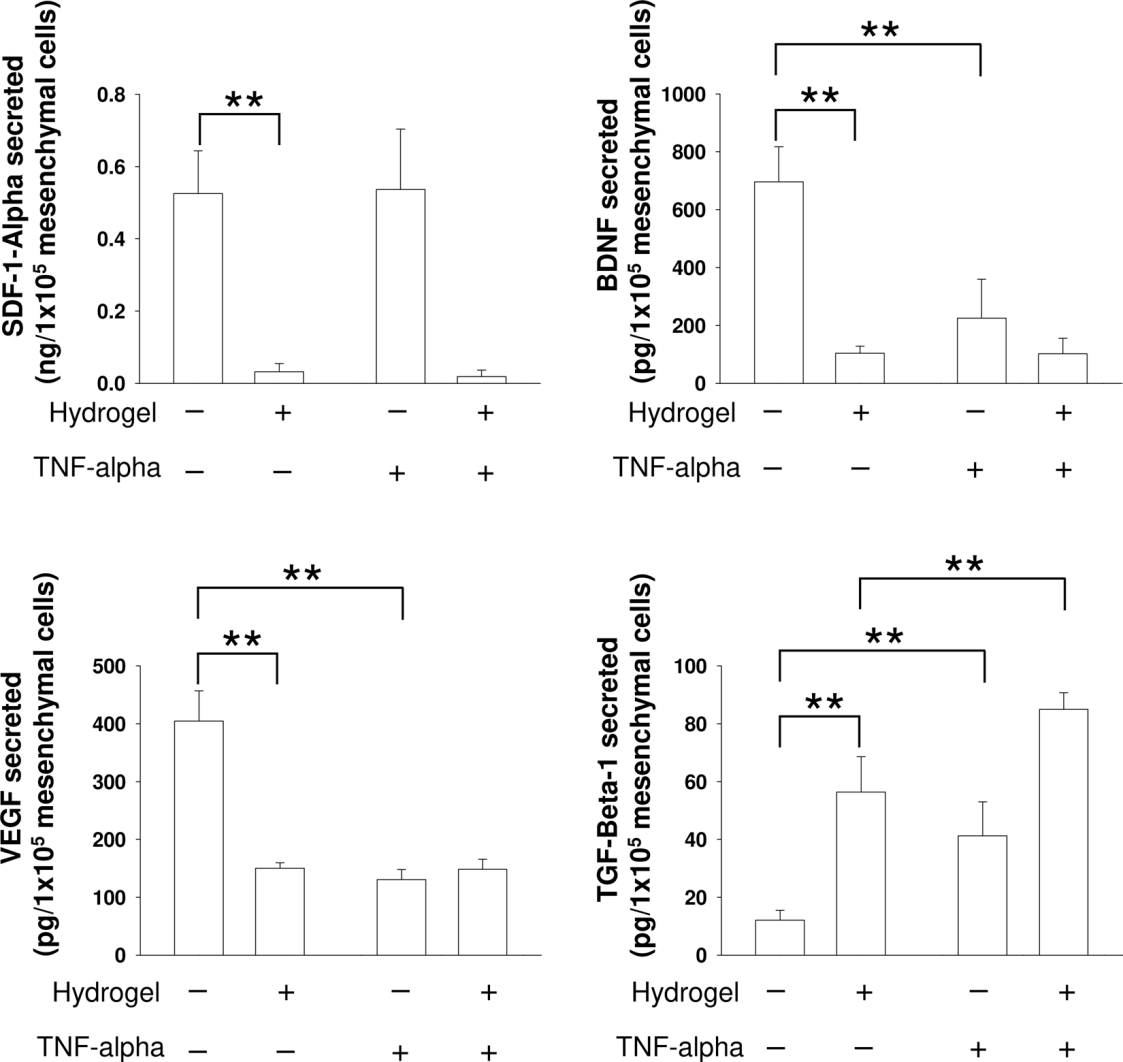
Analysis of the neurosecretome of MSCs encapsulated in silk fibroin hydrogels. Extracellular SDF-1, BDNF, VEGF and TGF-Beta-1 levels determined by ELISA in non-encapsulated MSCs and encapsulated MSCs in silk fibroin hydrogels. In parallel experiments, TNF-alpha was added to examine the responsiveness of MSCs to signals from the extracellular environment. Note the reduced secretion of the majority of factors examined in encapsulated MSCs. This was not the case for TGF-Beta-1 whose release was strongly augmented. The data are shown as mean ± the SEM with a minimal of three independent experiments and 12 samples per group (Student’s t-test; **p < 0.01).

The neurotherapeutic potency of MSCs has been attributed to their secretome activity^3,33^. In light of our results, it is reasonable that cell encapsulation in SF hydrogels might affect the MSCs secretome. We analyzed the MSCs release of several factors involved in neuroinflammation and brain plasticity after cerebral damage. The release of BDNF, SDF-1 and VEGF was severely reduced from encapsulated MSCs (Fig. 4). However, the secretion of the anti-inflammatory factor TGF-Beta-1 was substantially increased. We also examined the secretory ability of MSCs in response to the application of TNF-alpha, a molecule that is overexpressed in response to brain injury and has pleiotropic inflammatory roles. TNF-alpha is a typical molecule characteristic of the post-injury, neuro-inflammatory cerebral tissue. Exposure to this factor reduced the secretion of BDNF and VEGF, augmented the release of TGF-Beta-1 and did not change the release of SDF-1 from non-encapsulated MSCs. By contrast, in encapsulated MSCs, TNF-alpha did not modulate the secretion of BDNF, VEGF and SDF-1, while it significantly increased the secretion of TGF-Beta-1 **(**Fig. 4**)**. With the exception of TGF-Beta-1, our findings suggest that this biomaterial prevents the negative regulation of TNF-alpha on BDNF and VEGF secretion, which were reduced in non-encapsulated MSCs exposed to TNF-alpha.

**Figure 5:**
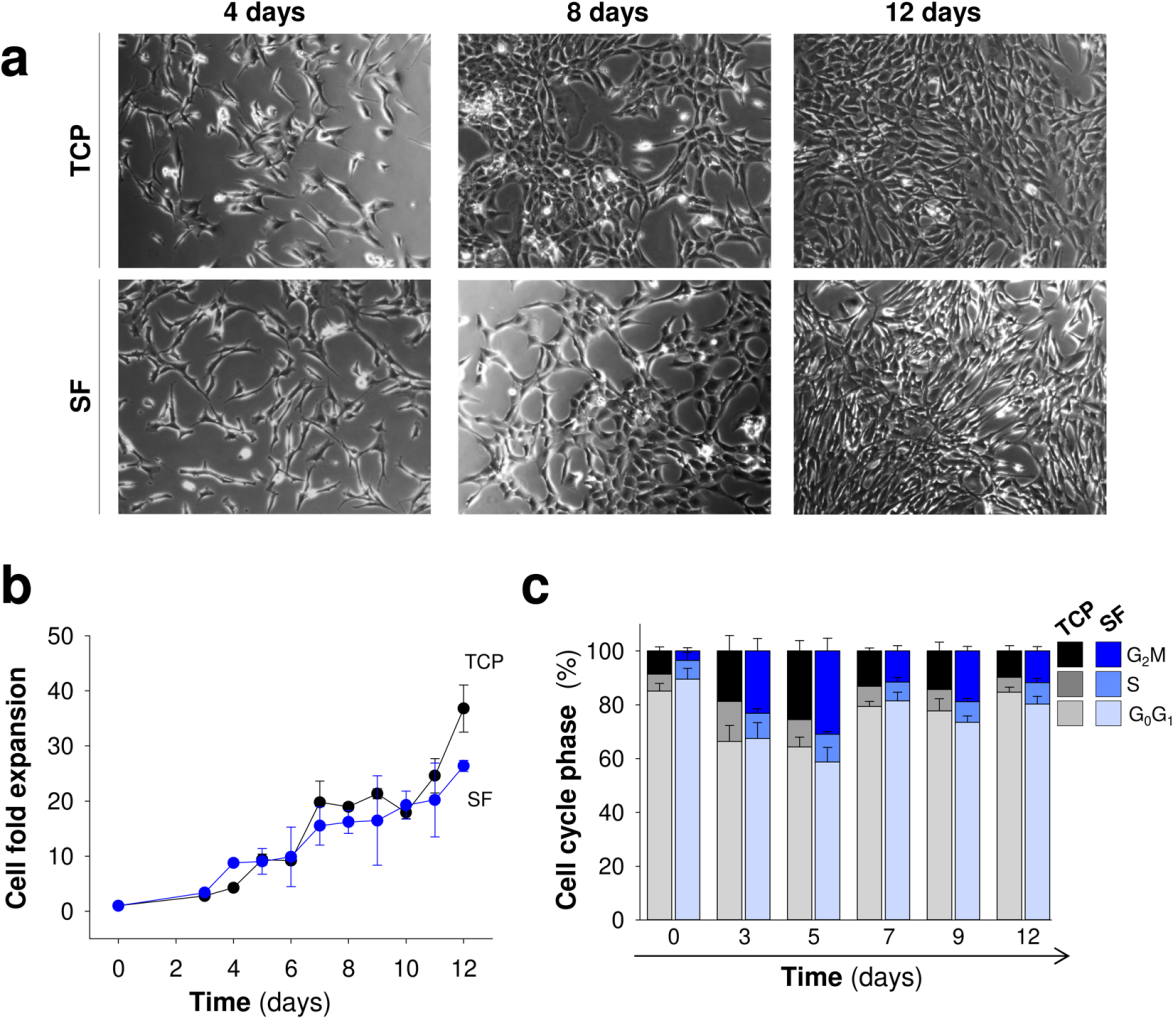
Proliferation of Mesenchymal stem cells in silk fibroin films. (**a**) Representative images of MSCs growing on plastic (TCP) and silk fibroin (SF) at different time points after seeding (scale bar: 200 μm). (**b**) Cell fold expansion over TCP or SF after time in culture. (**c**) Percentage of cells in each cell cycle phase (G_0_/G_1_, S and G_2_/M) across time in culture. The data in (**b**,**c**) are shown as mean ± the SEM of three independent experiments in triplicate (9 samples in total per group and temporal point).

**Figure 6:**
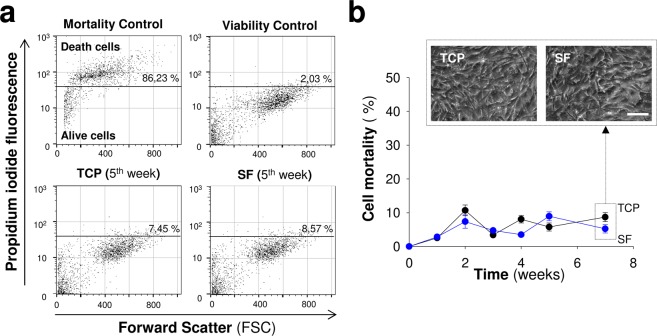
Short- and long-term viability studies of mesenchymal stem cells in contact with silk fibroin films. (**a**) Representative flow cytometry dot plots to illustrate the relationship between propidium iodide fluorescence intensity (a marker of late apoptotic and necrotic cells) and cellular size (Forward Scatter) in MSCs over TCP or silk fibroin. (**b**) Percentage of dead cells across time after seeding. The insets in the top part show representative microscopy images of MSCs grown over TCP or silk fibroin at seven weeks of culture (Scale bar: 100 μm). The data are shown as mean ± the SEM of three independent experiments with 9 samples per group and temporal point.

We cultured cells on SF films to assess the influence of fibroin on cell adhesion, survival and proliferation and discriminate the role of composition and structure in the more complex gel format. In our hands, the short- and long-term stability of fibroin films for cell culturing could be possible at higher polymer concentrations (6%) and only when this material was solubilized in Hexafluoro-2-propanol (HFIP). SF films prepared from aqueous solutions had little stability and most of the material (ranges assayed: 2, 4, 6 and 8%) was completely fragmented without covering with uniformity the surface of TCP wells used for the MSCs cultures (data not shown). Therefore, a 6% fibroin solution in HFIP was selected to study the interaction of MSCs with this specific biomaterial format. We explored the proliferative ability of MSCs in contact with SF films or TCP. Across time, MSCs fold expansion was similar on plastic and SF films, and MSCs showed a fibroblastic-like morphology in both culture conditions (Fig. 5a,b).The lack of proliferation differences correlated with the normal cell cycle detected by flow cytometry (Figs 5c and S2). The percentage of cells in each cell cycle phase (G_0_/G_1_, S and G_2_/M) was similar between plastic and SF conditions. The cultures reached maximal confluency on days 11–12, as the percentage of cells in S and G_2_/M phases decreased while those in the G_0_ quiescent state increased. Because cell proliferation is an indirect read-out of survival, we examined the short- and long-term effects of the biomaterial on MSCs survival. To this end, we treated the MSCs cultures with mitomycin C to arrest the cell cycle and stop proliferation, thus inducing prolonged quiescence^34^. In this condition, we maintained MSCs in contact with the biomaterial beyond seven weeks (Fig. 6). Across the time the cell mortality examined by flow cytometry and propidium iodide staining was near or below 10% in both, TCP and SF conditions **(**Fig. 6**)**. Thus, SF films at 6% did not severely impair cell survival or proliferation, a fact that contrasted with the impaired adhesion and growth of MSCs seeded on the top of SF hydrogels of 2%. The normal growth of MSCs in films was not due to the degradation of fibroin since this biomaterial at this concentration (6%) was uniformly covering the wells after study (seven weeks, data not shown).

**Figure 7:**
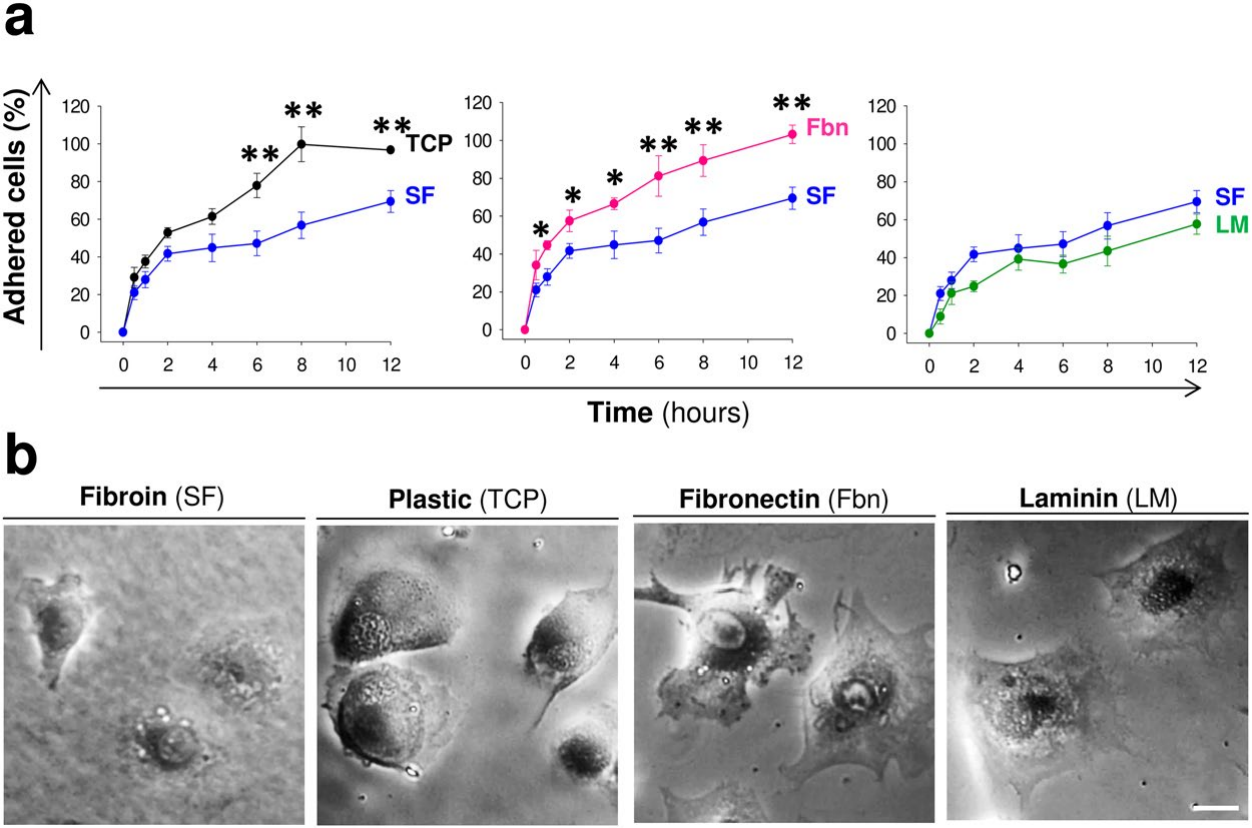
Cell adhesion kinetics. (**a**) Temporal course of MSCs adhesion over SF (blue circles), TCP (black circles), fibronectin (pink circles) and laminin (green circles). The data are shown as mean ± the SEM of three independent experiments with 9 samples per group and temporal point. The asterisks indicate significant differences between silk fibroin and the other materials tested (two-way ANOVA followed by Tukey’s test; *p < 0.05; **p < 0.01). (**b**) Representative images of MSCs adhered over SF, fibronectin, laminin and plastic 24 hours after seeding (Scale bar: 25 μm). At this early time point the cells in contact with SF showed a more spheroid morphology with less cytoplasmic extensions compared to those in contact with TCP, fibronectin or laminin.

Cell adhesion to the extracellular matrix (ECM) is a general aspect of cellular physiology that control processes such as cell spreading, survival, proliferation, differentiation and migration. Interaction between cells and extracellular proteins, typically through integrins and ECM proteins such as fibronectin or laminin, are essential for normal communication between cells or with the extracellular environment^35^. Early MSCs contact with different substrates including SF was explored by stick-wash assays at different time points after initial seeding. In these studies the MSCs took longer to adhere to SF and laminin than the other two substrates examined (fibronectin and TCP). Twelve hours after seeding the proportion of adherent cells was significantly lower in the SF and laminin groups **(**Fig. 7a**)**. Although by 24 hours post-seeding most of the cells were attached to all substrates examined, the MSCs in contact with SF had a spheroid-like shape with apparently less cellular processes (Fig. 7b). However, this visual difference did not translate to proliferation abnormalities, and cell morphology was normal at later time points (Fig. 5a).

**Figure 8:**
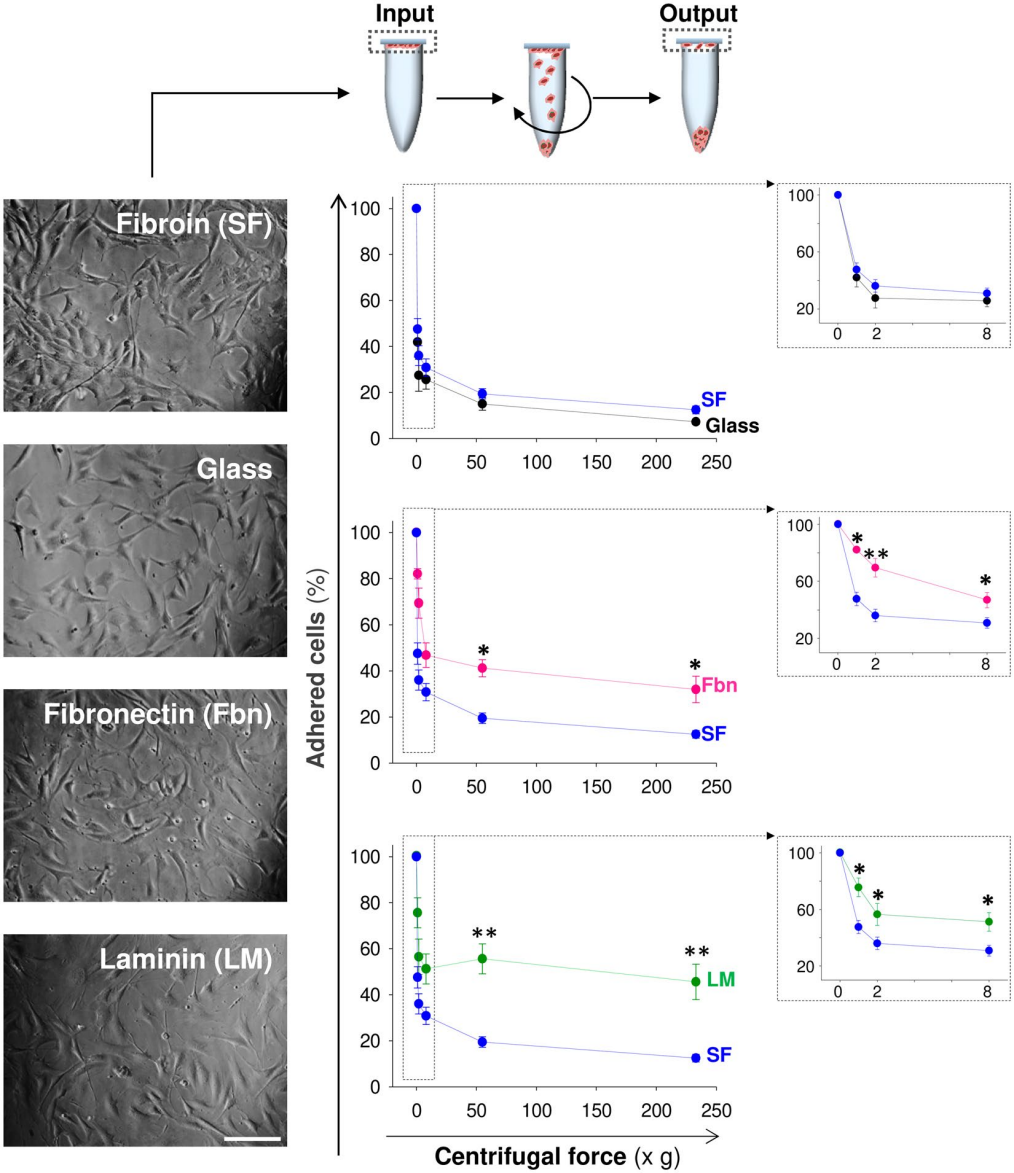
Changes in firm adhesion of mesenchymal stem cells seeded on silk fibroin films. In the top, scheme to illustrate how the intensity of cell adhesion over the different materials was estimated. First, a known number of MSCs (input) was seeded on coverslips coated with different materials. After 48 hours in culture, the coverslips were flipped upside down and placed over Eppendorf tubes filled with PBS. The different samples were centrifuged at variable centrifugal forces, and the number of cells still adhered to the coverslips was counted (output). On the left, representative images of MSCs seeded on different materials before centrifugation (Scale bar: 200 μm). On the right, relationship between the centrifugal force and the percentage of cells adhered on glass (black circles), SF (blue circles), fibronectin (pink circles) and laminin (green circles). The insets in the right part of the figure show the relationship between the centrifugal force and the percentage of adhered cells at low centrifugal force ranges (0–8 xg). The data are shown as mean ± the SEM of six independent experiments with 18 samples per group and temporal point. In the figure, the asterisks denote significant differences between SF and the rest of materials (two-way ANOVA followed by Tukey’s test; *p < 0.05; **p < 0.01).

Conventionally, ECM composition and mechanics impair the strength of cell adhesion and rate of spreading. Our previous adhesion study did not provide information on the intensity of MSCs adhesion to SF. We performed inverted centrifugation assays to quantify the strength of adhesion based on cytoskeleton changes following initial binding and receptor-ligand affinity with the different biomaterials tested^28^. After 48 hours in culture, the MSCs in contact with borosilicate glass coated with SF, laminin or fibronectin showed a fibroblastic-like shape **(**Fig. 8**)**. The MSCs also had a normal morphology on borosilicate glass alone, which was used as a control for theoretically lower cell attachment **(**Fig. 8**)**. Thus, the spheroid aspect (less fibroblastic) of MSCs in contact with SF at 24 hours after culture **(**Fig. 7b**)** was merely transitory. At low and high centrifugation values in this study, more cells remained attached to fibronectin and laminin than to SF or glass, suggesting that the strength of adhesion was lower on SF **(**Fig. 8**)**. Therefore, although SF films did not exert deleterious effects on MSCs survival and proliferation, cell attachment was impaired in initial kinetics and strength of adhesion.

## Discussion

Tissue engineering demands stem cells with therapeutic efficacy, low immunogenicity and minimal side effects. MSCs meet most of these requirements, as supported by their extensive use in the preclinical and clinical settings^36^. MSCs have been used in combination with different biomaterials for bone and cartilage regeneration, but their use with scaffolds to treat neurological disorders is more limited. Previous studies suggested that the neuro-therapeutic benefits of MSCs are mostly based on their secretome activity. MSCs-released factors prevent the deterioration of the injured brain by modulating host inflammation and immune-response and stimulating endogenous neurogenesis^16,18^.

In our study, good survival of MSCs inside SF hydrogels (2%) was accompanied by abnormal cytoskeletal rearrangements and reduced cell proliferation, which caused substantial changes in the secretome. In contrast to MSCs engrafted inside hydrogels, the survival of MSCs was strongly reduced on the top of 2% hydrogels, a fact that contrasted with the apparent normal growth observed for MSCs seeded on the top of SF at higher concentrations (6%). At the same polymer concentration of 2%, the MSCs survival rate was superior inside the hydrogels than on the top of the material, probably because the cellular confinement (cell-trapping) within the hydrogels counteracted the poor adherence of MSCs to SF, avoiding the strong cell detachment of MSCs seeded on the top of hydrogels. Although the distinct nature of biomaterial surface might contribute for differences in MSCs adhesion on the top of SF hydrogels and films, our results suggest that the lack of cellular adherence and expansion inside 2% SF hydrogels is probably due to the combination of two factors; polymer concentration and mechanical restrictions that might prevent cellular attachment and spreading.

MSCs have been grown in other biomaterial scaffolds such as alginate^37,38^, collagen^39^, Matrigel^40^, methylcellulose^41^ and nanofibrillar cellulose^42^. In the majority of biomaterials, the survival and proliferation of MSCs were not substantially impaired when these cells were grown on the surface of these polymers. For example, normal attachment and cell growth has been reported for MSCs on polyethylene glycol dimethacrylate hydrogels^43^, gelatin-alginate scaffolds^44^ of 3D porous SF/ hydroxyapatite (HAP) hydrogels^45^. Additionally, normal attachment, spreading, spindle-like morphology, survival and multipotent differentiation have been reported for MSCs in contact with composites of SF formed with hyaluronic acid^46,47^, 2-hydroxyethyl methacrylate^48^ and chitosan^49,50^. In this context, our results are aligned with various studies that analyzed MSCs fitness over SF. These reports have shown that MSCs attachment and survival positively correlate with SF stiffness and polymer concentration, because stiffness polymers provide better mechanical stability for cell adhesion and growth^51,52^.

It is well established fact that the relatively good fitness of MSCs seeded on different biomaterial scaffolds and hydrogels changes dramatically when these cells are grown inside the different materials, which agrees with our observations. It has been reported that MSCs encapsulated in distinct hydrogels remain alive, but they lost their typical spindle-like shape and are less elongated. This may be related to deficient cytoskeletal rearrangements that induce the quiescent state when proliferation is diminished or even abolished. For example, MSCs with spherical aspects and scarce proliferation survive into methacrylated hyaluronic acid hydrogels^53^ and SF-gelatin crosslinked with tyrosinase^54^. In other examples, MSCs had spherical round morphologies inside nanofibrillar cellulose hydrogels and cell viability was moderately affected at higher (0.5%) polymer concentrations^42^. Similarly, pluripotent stem cell-derived MSCs encapsulated into PEGDA hydrogels survive for several days with spherical morphologies different from their natural spindle-like aspects when grown on the surface of this hydrogel^55^. This is in line with the possibility that MSCs confined in the interior of these hydrogels have cytoskeletal aberrations incompatible with normal growth. In relation to silk, a previous study reported that MSCs survive for up to 10 days in culture when encapsulated in SF hydrogels while they have a moderate proliferation rate^56^. Parallel studies have examined the effect of SF stiffness on MSCs survival, finding that MSCs viability inside SF hydrogels is drastically reduced at polymer concentrations higher than 4–6%^31,32^.

In addition to stiffness, the composition of biomaterials affects MSCs survival and growth inside hydrogels. For example, MSCs were unable to expand inside gellan gum hydrogels and had spherical non-spindle-like morphologies with lower viability and less cellular processes than MSCs grown in the same biomaterial functionalized with the GRGDS fibronectin-derived peptide, where the MSCs acquired the characteristic spindle-like shape^57^. In another example, MSCs survived in hydrogels formed by peptides of 12 amino acids in length containing the RGD sequence (fibronectin). Specifically, this hydrogel supported cell adhesion, survival and differentiation towards cardiac cells after *in vivo* transplantation to enhance cardiac function after myocardial ischemia^58^. Both studies are interesting because they highlight the importance of using natural extracellular motifs to enhance cell survival and growth beyond other limiting factors such as polymer concentration and excessive confinement. An injectable hydrogel based on a composite of hyaluronic acid and alginate was used to encapsulate MSCs and induced better survival and expansion rates in formulations containing the double amount of alginate than hyaluronic acid^59^. Under this condition, MSCs integrated in these hydrogels improved functional outcomes after traumatic brain injury^59^. In another study, MSCs of cardiac origin survived for 14 days within SF hydrogels functionalized with albumin-perfluorohexane microspheres^60^. These microspheres increased the porosity of SF hydrogels enhancing cell viability^60^. This shows how restricted porosity (excessive confinement) might influence attachment and proliferation to prevent the movement, spreading and elongation of MSCs cultured inside hydrogels.

An aspect that deserves consideration is the influence of serum in our assays. Cellular attachment in the absence or presence of serum on different substrates is dependant of the specific cell type^61^. In the case of MSCs in contact with tissue culture polystyrene, no strong differences in attached cell numbers or spreading area were observed between serum and non-serum conditions^61^. This can be related with the fact that MSCs secrete different components of ECM such as fibronectin, laminin or collagen^62,63^. The adhesion mechanisms of MSCs to SF films are difficult to decipher due the lack of adhesion motifs in the SF sequence, motifs like the RGD sequence that is present in ECM proteins such as fibronectin. The lack of RGD motifs on SF might partially justify the better adhesion of MSCs on fibronectin and laminin observed in our study. In relation with literature, we can not discard that adhesion of MSCs to SF could be produced directly via electrostatic interactions. However, MSCs adhesion to SF might be mostly mediated by factors released from MSCs (fibronectin, laminin), factors that are also present in serum, which can be adsorbed on the substrate (SF) mediating cell attachment. In our work, we tested cell adhesion under serum conditions to establish a link with the secretome analysis and our previous *in vivo* study^23^, where MSCs implantation with SF hydrogels were performed in the brain striatum, exposed to extracellular matrix components regularly found in serum. We also sought to establish a comparison with previous studies that examined MSCs adhesion and growth on the top or inside SF under serum conditions^31,32,52^. Specific research is needed to clarify the influence of non-serum environments in the cellular adherence and secretome activity of MSCs in contact with SF.

The neuroprotective and neurorepair mechanisms of MSCs have been attributed to their secretome. However, few studies have examined the influence of different polymers on this aspect of MSCs function. In one study, MSCs survived on methylcellulose or alginate hydrogels^64^, although the number of encapsulated cells did not change across 4 weeks of study. Under this condition, MSCs released indoleamine 2,3-dioxygenase, prostangladine E2 and hepatocyte growth factor, which are known immunomodulatory and anti-inflammatory biomolecules. Interestingly, the secretion of these 3 factors from MSCs was augmented after stimulation with inflammatory molecules such as TNF-alpha and Interferon-gamma^64^. The secretory ability of stem cells has been enhanced through engineering and preconditioning strategies. For example, MSCs genetically modified to overexpress BDNF, survived and proliferated when encapsulated in self-assembling peptide RADA16-PRG hydrogels^65^. The release of BDNF was dependent of the PRG sequence which contains repeats of the RGD domain. However, in RADA16 hydrogels that lack the PRG sequence, the expansion of MSCs was lower and the release of BDNF was significantly reduced. The study of Silva and co-workers is very interesting^57^. Although specific factors were not measured directly in that study, the conditioned media from MSCs encapsulated into gellan gum hydrogels did not support the *in vitro* survival of neuronal primary cells. This contrasted with the beneficial effect exerted by conditioned media from MSCs encapsulated in the same biomaterial hydrogel bioengineered with the extracellular matrix-derived peptide GRGDS^57^. Thus, the existence of adhesion motifs (naturally or synthetically incorporated) in different biomaterials might enhance the release of factors from the different stem cells encapsulated in them. In our case, the normal survival of MSCs inside SF gels was accompanied by substantial reductions in the release of BDNF, SDF-1 and VEGF that could be related to the absence of adhesion motifs and particular mechanical and environment properties of 3D silk gels. By contrast, the secretion of TGF-Beta-1 was considerably augmented after MSCs encapsulation. We analyzed the secretion of these factors because of their strong repercussions on inflammatory, angiogenic and neurogenic scenarios after cerebral damage^66–68^. For example, TGF-Beta-1 is a known anti-inflammatory and angiogenic molecule. Several studies have illustrated that treatment with TFG-Beta-1 enhances recovery after cerebral damage by polarizing microglia towards the anti-inflammatory phenotype. In our hands, this increasing secretion of TGF-Beta-1 might tentatively and partially explain the improved recovery of mice after stroke treated with MSCs encapsulated in SF hydrogels^23^. The mechanisms of enhanced TGF-beta-1 secretion from encapsulated MSCs are largely unknown but several possibilities exist for further analysis. First, abnormalities of actin cytoskeleton and impaired cellular adhesion to generate force and/or to provide mechanical resistance might be responsible for changes in TGF-Beta-1 expression and secretion from encapsulated MSCs^69^. A second possibility is related with oxidative stress. Accumulation of reactive oxygen species (ROS) has been related with enhanced TGF-Beta-1 expression and secretion in different cell types^70^. Although most of the MSCs were alive (~80–90% survival), adhesion and proliferation deficits were observed in MSCs growing inside SF hydrogels. These deficits may reflect the existence of several cellular stressors including abnormal accumulation of ROS. At this point it is unknown for us whether ROS levels might be modified in encapsulated respect non-encapsulated MSCs, establishing a cause/relation effect between ROS content and TGF-Beta-1 hypersecretion in encapsulated MSCs. A third possible mechanism is related with the existence of a hypoxic microenvironment inside SF hydrogels. Hypoxic preconditioning stimulates the expression and secretion of several growth factors and cytokines from MSCs, including TGF-Beta-1^71^. In one interesting study^72^, the existence of a hypoxic microenvironment due to limited oxygen diffusion has been proposed as a main mechanism for the enhanced secretion of TGF-Beta-1 in MSCs growth as 3D spheroids in comparison with MSCs cultured as adherent monolayers. Although the *in vitro* secretion of BDNF, VEGF and SDF-1 was reduced in encapsulated MSCs, MSCs have better survival in the brain after encapsulation in SF hydrogels^23^. This creates a complex scenario in which a deficient secretion of these factors might coexist along with persistent *in vivo* factors release from encapsulated MSCs. By contrast, we also examined the effect of TNF-alpha on the MSCs secretome. It have been reported that TNF-alpha is upregulated after brain injury, and it has pro- and anti-inflammatory roles^73,74^. In encapsulated MSCs TNF-alpha failed to modulate the secretion of three of the four factors examined. However, the effects of TNF-alpha were largely variable on non-encapsulated MSCs. With the exception of TGF-Beta-1, the lack of change in BDNF and VEGF secretion by encapsulated MSCs induced by TNF-alpha does not seem to be related with deficits of TNF-alpha entry into the hydrogel and probably depends of impairment of downstream TNF-alpha pathways.

Although an excessive confinement of MSCs might be detrimental for MSCs growth and function after *in vivo* transplantation, a hydrogel with less restricted porosity would probably positively impact cell proliferation. This would be at the expense of higher uncontrolled cell migration in to and out of the hydrogel, facilitating the entry and immune-attack of resident cells on transplanted cells and accelerated biomaterial degradation. Therefore, we believe that a structural and functional component with the best balance between hydrogel porosity and cellular activity should be found. Although films might offer significant advantages for MSCs growth and expansion, hydrogels might fill amorphous cavities resulting from injury and can be better for intracerebral cell transplantation applications. In cerebrovascular disorders an interesting application of SF films might be related with control of drug delivery by bypassing the blood-brain-barrier^75^. Although our research is aimed towards neurotherapeutic potential of MSCs, our results might have repercussions in other areas of tissue engineering.

## Conclusions

Collectively, in this study we determined that stem cells and progenitors of mesenchymal origin (MSCs) survive for several days when encapsulated in 3D silk fibroin hydrogels. We provided evidence that several neuroprotective and neuroregenerative factors are released through this biomaterial, although their secretion was reduced compared to that from non-encapsulated MSCs. This was not the case for the anti-inflammatory factor TFG-Beta-1 whose release was unexpectedly and substantially augmented. In this case, the encapsulation of MSCs in silk fibroin provided an additional engineered advantage to oversecrete this specific anti-inflammatory factor. Here we set the precedent for continued analysis of the MSCs secretome in native or functionalized silk fibroin or in composites of this material with other biocompatible polymers. The secretome of multipotent and pluripotent stem cells constitute a mechanism to sustain recovery in many cerebrovascular and neurodegenerative disorders treated with cell-therapy. In the preclinical and clinical settings, it is essential to identify the molecule or group of molecules responsible for the reported therapeutic effects, define the relationship of the local concentration between secreted factors, and elucidate the temporal window of application and dynamics of release. This progressive knowledge will help to design improved engineering biopolymers with optimized neurosecretomes for repairing the injured brain.

## Supplementary information

Supplementary material
