# Supplementary material for: Evaluation of Neurosecretome from Mesenchymal Stem Cells Encapsulated in Silk Fibroin Hydrogels
Source: Sci Rep. 2019 Jun 19;9:8801. doi: 10.1038/s41598-019-45238-4 (PMC6584675; doi:10.1038/s41598-019-45238-4)
Supplement: Supplementary file 1 — Supplementary material [file 41598_2019_45238_MOESM1_ESM.doc]

**SUPPLEMENTARY INFORMATION**

**EVALUATION OF NEUROSECRETOME FROM MESENCHYMAL STEM CELLS ENCAPSULATED IN SILK FIBROIN HYDROGELS**

Yolanda Martín-Martín, Laura Fernández-García, Miguel H. Sánchez-Rebato, Núria Marí-Buyé, Francisco J. Rojo, José Pérez-Rigueiro, Milagros Ramos, Gustavo V. Guinea, Fivos Panetsos, and Daniel González-Nieto

Supplementary information consists of:

1. Supplementary Methods
2. Supplementary Figure 1- Expansion and multipotent differentiation of Mesenchymal Stem Cells
3. Supplementary Figure 2- Flow cytometry strategy to analyze cell cycle in Mesenchymal Stem Cells growing over silk fibroin films

**Supplementary methods**

*1. Functional characterization of multipotent mesenchymal stromal cells*

The multipotent differentiation of MSCs was assessed as previously described. Briefly, 0.5-2x10^6^ cells were seeded and incubated in conditioned culture media containing IMDM with 20 % FBS and 0.1μM dexamethasone. This medium was supplemented with 0.25 mM ascorbic acid and 10 mM 2-glycerolphosphate for osteoblast differentiation; 100 μM indomethacin and 5 μg/ml insulin for adipocyte differentiation; or with 10 ng/ml TGF-β1 to obtain chondrocytes. The cell medium was changed by weekly hemidepletion. After 3-4 weeks in differentiation medium the osteoblasts, adipocytes and chondrocytes were identified by positive staining for alkaline phosphatase, oil red and toluidine blue respectively.

*2. Cell distribution in hydrogels*

To examine the cellular distribution on SF hydrogels, a total of 1.5x10^5^ MSCs were mixed with 500 µl of a sonicated SF solution in a pre-gel state (complete MSCs medium) and plated on 24-wells plates (BioLite, Thermo Scientific). After gelation was complete, a 5mm-diameter puncher was used to extract cylindrical molds of 9 mm high x 5 mm in diameter (~ 176 mm^3^ in total volume). These cylindrical molds were cultured in new 24-wells plates in complete medium for 2 or 4 weeks. Live cells were identified by incubation with calcein-AM. Cellular distribution inside the hydrogel was analyzed by cell counting in several sections across the longitudinal axis of the cylindrical molds. Each cylindrical hydrogel was cut with a rectangular glass coverslip to obtain three longitudinal pieces. Each longitudinal piece was divided in 12 volume parts (1 mm wide x 0.75 mm length x 0.4 mm thickness; each volume part was 0.3 mm^3^) along the longitudinal axis. Cell counts were obtained for each volume part and in triplicate (three longitudinal pieces). These values were used to calculate the equivalent cell content of each volume part (~ 14.6 mm^3^) in the cylindrical mold (total volume ~ 176 mm^3^). The identification of calcein-positive cells was performed under a fluorescence microscope (Leica DMI3000, Nussloch, Germany) coupled with a Leica DFC340FX camera.

*3. Proliferation studies, analysis of cell cycle by flow cytometry*

A total of 5x10^3^ MSCs were seeded over SF films or TCP (24 well plates, BioLite, Thermo Scientific). Cell fold expansion was estimated by daily counting starting at third day after seeding. After removal the culture medium, the cells were washed and incubated for 5 minutes at 37 ° C with 100 µl of trypsin-EDTA 0.5% (gibco® life technologies) to cause cell detachment. The action of trypsin was blocked adding 10 µl FBS and the recovered cells were counted in a Neubauer chamber. For cell cycle studies a total of 20x10^3^ MSCs were cultured in complete medium for 24 hours. Cell synchronization was induced by serum starvation during 72 hours. Then, MSCs were reincubated with complete culture medium and detached with trypsin at different time points after synchronization. The recovered cells were fixed for 1 hour on ice with cold ethanol (70%). The fixed cells were centrifuged and washed with PBS twice. Cells were incubated for 15 min (37ºC) with 5 µg ribonuclease (100µg/mlRibonucleasa A Solution; Sigma Aldrich; Cat# R4642) and for 20 min (room temperature) with 7-actinomicin D (2 µg/ml; 7-ADD; lifeTecnologies; Cat# A1310). Flow cytometry was carried out in a BD FACSCalibur (Becton, Dickinson and Company) on the FL2 channel (excitation at 488 nm and emission at 585 nm). The events were acquired through OS 9.2.2, CellQuest Pro 4.0.2 software. The different cell cycle phases (G_0_/G_1_, S, and G_2_/M) were identified and estimated by a combination of gating selection and 7-ADD fluorescence (**Supplementary Figure S2**) using FlowJo software (FlowJo® v10.2, LLC, Ashland, Oregón).

**References**

1. Fernandez-Garcia, L. et al. Cortical Reshaping and Functional Recovery Induced by Silk Fibroin Hydrogels-Encapsulated Stem Cells Implanted in Stroke Animals. Front. Cell. Neurosci. **12**, 296; [10.3389/fncel.2018.00296](https://doi.org/10.3389/fncel.2018.00296) (2018).

2. Gonzalez-Nieto, D. et al. Connexin-43 in the osteogenic BM niche regulates its cellular composition and the bidirectional traffic of hematopoietic stem cells and progenitors. Blood **119**, 5144-5154 (2012).

**Supplementary figures**

**Figure 1. Functional characterization of mesenchymal stem cells.** (A) Representative images of bone marrow mesenchymal stem cells and progenitors (MSCs) growing on the top of plastic at different passages after bone marrow extraction and isolation. (B) Multipotent differentiation of mCSs cultures towards osteoblasts, adipocytes and chondrocytes.

**Figure 2. Cell cycle analysis of mesenchymal stem cells growing on silk fibroin films.** (A) Scheme illustrating the different cell cycle phases. From a quiescent state (G_0_) cells can enter into G_1_ to initiate mitosis. During G_1_ mRNA and protein synthesis is produced. At the end of G_1_ DNA synthesis is initiated (S phase) and cell growth occurs (G_2_) until genetic material is distributed between both cells coinciding with cell division (M). The different cell cycle phases might be distinguished through flow cytometry analysis and 7-aminoactinomycin D (7-AAD) fluorescent load. (B) Flow cytometry strategy to examine the proportion of cells in each cell cycle based on cell size (forward scatter), complexity (side scatter) and 7-ADD fluorescence level. (C) Representative histograms of 7-ADD fluorescence of MSCs proliferating on culture plastic (TCP) or silk fibroin films at day 0 and 5 after MSCs seeding.
